# Self-perception, quality of life and ease of catheterization in patients with continent urinary diversion with the mitrofanoff principle

**DOI:** 10.1590/S1677-5538.IBJU.2019.0388

**Published:** 2020-07-31

**Authors:** Julián Chavarriaga, Nicolás Fernández, María A. O. Campo, John Bolivar, German Patiño, Jaime Perez

**Affiliations:** 1 Pontificia Universidad Javeriana Hospital Universitario San Ignacio Division of Urology Bogotá Colombia Division of Urology, Hospital Universitario San Ignacio, Pontificia Universidad Javeriana, Bogotá, Colombia; 2 Fundación Santa Fe de Bogotá Division of Urology Colombia Division of Urology, Fundación Santa Fe de Bogotá, Colombia; 3 University of Toronto Hospital for SickKids Division of Urology Canada Division of Urology, Hospital for SickKids, University of Toronto, Canada; 4 University of California Division of Urology San Francisco United States of America Division of Urology, University of California San Francisco, United States of America

**Keywords:** Urinary Diversion, Quality of Life, Urinary Bladder, Neurogenic

## Abstract

**Purpose:**

Continent urinary diversion (CUD) with the Mitrofanoff principle stands as an alternative to urethral catheterization by a route other than the urethra. The aim of the study was to determine self-perception of health-related quality of life (HRQoL), ease of catheterization and global and cosmetic outcomes in patient’s dependent on Mitrofanoff catheterization.

**Materials and methods:**

Records of all patients who underwent CUD with the Mitrofanoff principle between 2012 to 2018 were reviewed. Data were collected and analysed retrospectively from medical charts. We assessed HRQoL with the EuroQol EQ-5D-3L questionnaire, cosmetic and global satisfaction with a questionnaire designed by the reconstructive urology board and ease of catheterization with a Likert questionnaire adapted from the Intermittent Catheterization Difficulty Questionnaire (ICDQ) validated in patients reliant on retrograde CIC.

**Results:**

A total of 25 patients requiring CUD with the Mitrofanoff principle between 2012 and 2018 were assessed, the group was composed mainly of: appendiceal conduits 18 patients (72%) and 7 ileal conduits (Yang-Monti) and three of those requiring Casale (Monti Spiral) and 1 a double Monti technique. Median follow-up was 57 months, median age was 30 years. Visual Analogue Scale (VAS) of the EQ-5D-3L reported a Global health score of 86.5%. Fifty nine percent of the patients had no pain or bleeding with catheterizations. Regarding global satisfaction and cosmetic perception 91% were satisfied with their CUD.

**Conclusions:**

CUD is associated with good HRQoL, global satisfaction, ease and painless catheterization, adequate self-perception of cosmetic outcomes and a low complication rate, remaining a safe and viable option.

## INTRODUCTION

**Figure 1 –:**
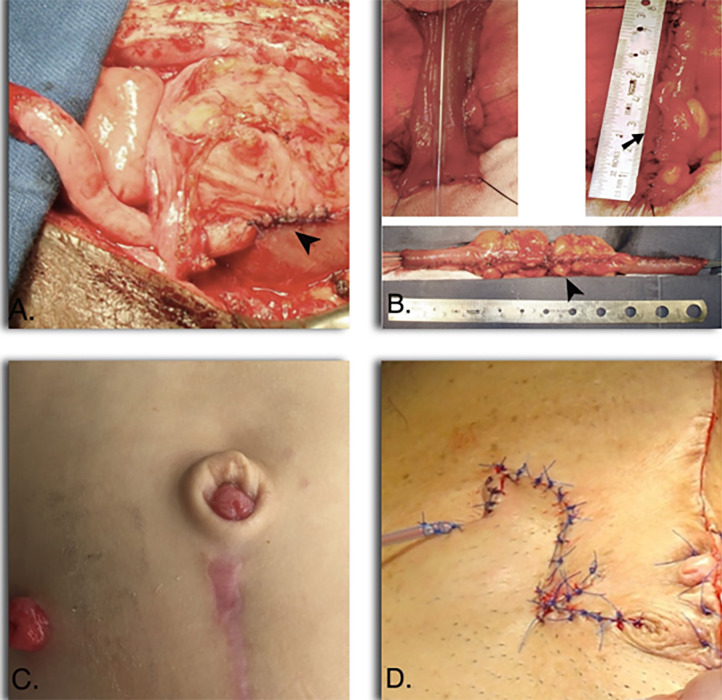
A) Appendicovesicostomy with the Mitrofanoff principle showing an anti-reflux extravesical reimplantation technique (arrow head) as the anti-incontinence mechanism. B) Continent urinary diversion with the Mitrofanoff principle using an ileal segment for a transverse ileal tube (Yang-Monti) (arrow) and a double tube (Monti technique) (arrow head). C) Umbilical Stoma in a patient who underwent an Appendicovesicostomy and Malone antegrade continence enema (MACE) procedure. D) V-Quadrilateral-Z (VQZ) plasty stoma in a patient with a complex urethral stricture.

Paul Mitrofanoff described the “trans-appendicular continent cystostomy” in 1980, 8 years after Lapides described the clean intermittent catheterization technique (CIC) (1, 2). Both approaches have revolutionized the management of neurogenic bladder (1). Santiago Triana in 1947 have already described a trans-appendicular continent reservoir, replacing the bladder with an isolated segment of the cecum, despite being the first author to describe it, his technique never became as popular as the Mitrofanoff principle (3). The CUD with the Mitrofanoff principle described a new concept where the bladder could be emptied by a route other than the urethra. This was of remarkable importance specially when the urethra could not be used, or the patient would require lifelong CIC. Initially the appendix was the only segment of bowel used and the bladder neck was usually closed. As the appendix is not always available, different techniques have been described over time with other intestinal segments but based on the Mitrofanoff principle (3). Some variations are the transverse ileal tube (Yang-Monti), the double tube (Monti technique) and the Casale (Monti Spiral technique) (Figure-1). There has also been reports of conduits constructed with fallopian tubes, gastric segments, ureter (hydroureter) and even tubularized preputial transverse island flaps which have all been abandoned (1–3, 5).

Indications for CUD with the Mitrofanoff principle are neurogenic bladder with or without urethral lengthening, bladder neck closure or augmentation cystoplasty when required, complex urethral strictures due to location, non-viable urethral reconstructive surgery or previous brachy-radiotherapy, bladder dysfunction with intact urethral sensation (Congenital Obstructive Posterior Urethral Membrane (COPUM), Prune Belly syndrome, bladder or cloacal exstrophy-epispadias complex and idiopathic dysfunctional bladder). The same principle could be applied to the Malone Antegrade Continence Enema (MACE) for intractable constipation (1, 4, 5).

The use of retrograde CIC causes considerable changes in patient’s daily activities, modifying their social routine, professional activities and sexuality (6). In some cases, family members or caregivers who accompany the patient during treatment and are accountable for the performance of the CIC would also have an impact in HRQoL. Furmincelli, et al. evaluated 13 studies that reported on HRQoL in patients on CIC finding that patients on CIC presented lower QoL scores as well as their caregivers (6).

HRQoL in patients reliant upon Mitrofanoff catheterization had been reported to be good in most series (7, 8). Ease of catheterization had roughly been described in patients with CUD with the Mitrofanoff principle and had been more focused on the type of stoma (Umbilical, V-Quadrilateral-Z (VQZ) plasty (Figure-1) and V-Quadrilateral (VQ) flaps) being easy in most patients (5, 7). Cosmesis and satisfaction had been described to be satisfactory or excellent in most patients but doing emphasis on the type of stoma (1, 5, 7–9) The aim of this article is to determine HRQoL, ease of catheterization, and self-perception of global satisfaction and cosmetic outcomes in a subset of patients with CUD with the Mitrofanoff principle and to compare the results in patients with umbilical stomas and V-Quadrilateral-Z (VQZ) plasty stomas.

## MATERIALS AND METHODS

After IRB approval, IRB number 20190415755, records of all patients that underwent CUD with Mitrofanoff principle between 2012 to 2018 were reviewed. Data was collected and analysed retrospectively from medical charts. Each patient was invited to participate in our study and a fully signed informed consent was required to participate. A total of 22 patients out of 25 were contacted by phone (in order to get as many participants as possible we called each patient at least three times at different days or hours). For all patients that answered the phone-call, a cross-sectional design was used to evaluate HRQoL using the EuroQol EQ-5D-3L questionnaire. Approval from the EuroQol Research Foundation was granted. This is a 5-question health-related survey, each question with three possible answers assessing mobility, self-care, usual activities, pain or discomfort, anxiety and depression. Each of the five dimensions comprising the EQ-5D-3L descriptive system is divided into three levels of perceived problems (LEVEL 1: indicating no problem, LEVEL 2: indicating some problems, LEVEL 3: Indicating extreme problems). It also has a Visual analogue scale (VAS) to measure health status from 0 (the worst health you can imagine) to a 100 (the best health you can imagine) and it gives a EQ-VAS score (10, 11).

For pediatric patients it was a requisite that they were able to read and write in order to answer the questionnaire with adult assistance.

In order to assess cosmetic and functional outcomes we used a questionnaire designed by the members of the reconstructive urology division of the San Ignacio University Hospital (HUSI). This questionnaire included 5 questions with a YES/ NO answer. To evaluate ease or difficulty of catheterization we adapted with help of the functional urology, neuro-urology and reconstructive urology divisions of the HUSI, the Questionnaire for Intermittent Catheterization Difficulty Questionnaire (ICDQ) which was validated to evaluate difficulty with catheterization in patients reliant on retrograde CIC (13). The final Likert Questionnaire is made of 5 questions with 4 answer options for each question (0-Never, 1-Infrequent, 2-Frequent and 3-Always).

## RESULTS

Twenty-five patients requiring CUD with the Mitrofanoff principle between 2012 and 2018 were included in the study. Median follow-up was 57 months with an IQR of 9-84 months, median age was 30 years with an IQR of 5-76 years. Nineteen men and 6 women had surgery, the group was composed mainly of appendiceal conduits 18 patients (72%),7 ileal conduits (Yang-Monti) (28%), three of those requiring Casale (Monti Spiral) (12%) and one a Double tube (Monti technique). Diagnosis and indications for the procedure were complex urethral strictures in 12 (48%) patients, neurogenic bladder, 7 (28%), bladder or cloacal exstrophy and epispadias complex 4 (16%), one (4%) Casamassima syndrome and complex recto-vesical fistula in one patient (4%). Seventeen (68%) patients had a flank VQZ stoma and 8 (32%) had an umbilical stoma. Seven patients were pediatric (younger than 18 years-old), with a mean age in this subgroup of 8.5 years. Regarding complications, there were 4 major complications in our study. Two were appendiceal conduit necrosis. One case had partial necrosis of the conduit that was re-tailored and successfully reconstructed 6 months after the initial surgery and the other case was a complete loss of the conduit, The patient finally opted for a suprapubic catheter. Two patients had urinary incontinence. One due to failure of the antireflux mechanism, which was solved by endoscopic bulking agent injection. The other patient had incontinence due to intrinsic sphincteric deficiency that required bladder neck closure. Both patients were dry at the time of the study. Three patients presented stomal stenosis and none of them required revision and were managed with indwelling catheters. A summary is presented in Table-1.

**Table 1:** Revision and were managed with indwelling catheters.

Number of patients, *n*		25
Age (years)		Median 30, IQR (5-76)
Male:Female Rate		2.5:1
**Stoma**		
	Flank VQZ	17
	Umbilical	8
**Surgical Technique n (%)**		
	Appendicovesicostomy	18 (72)
	Ileal conduit (Yang- Monti)	6 ((24)
	Double Ileal Tube (Monti)	1 (4)
	Casale (Spiral Monti)	3 (12)
**Primary Diagnosis and indication for CUD n (%)**		
	Spinal dysraphism	1 (4)
	Exstrophy-Epispadias complex	2 (8)
	Cloacal abnormality	2 (8)
	Complex Urethral Stricture	12 (48)
	Neurogenic Bladder	6 (24)
	Recto-vesical fistula	1 (4)
	Other (Casamassima Syndrome)	1 (4)
Complications n (%)		4 (16)
Conduit Necrosis		2 (8)
Incontinence		2 (8)
Stomal Stenosis		3 (12)
Follow-up (Months)		Median 57, IQR (9-84)

Of the 25 CUD patients, we were able to contact 22 (88%). One of the patients who could not be contacted had died of a cause other than the surgery. The other two could not be reached at the phone-number registered in the medical chart. HRQoL was assessed using the EQ-5D-3L Questionnaire. We found that 95% of our patients had no problems with mobility. Ninety one percent had no problems with self-care, 77% were able to do their usual activities and of the 23% that had some problems, only one blamed the surgery to be the cause. Eighty six percent had no pain or discomfort in daily activities and 14% did mention some sort of discomfort or pain in daily activities. We confirmed with the patients that the pain referred was not related to the CUD. Regarding anxiety and depression, 59% did not consider themselves anxious or depressed, while 41% said to be moderately anxious or depressed.

EQ VAS of the EQ-5D-3 reported a Global health score of 86.5%. Sixty eight percent reported their global health score to be between 80100% and only one patient scored below 60%.

To evaluate ease of self-catheterization we use our own Likert Questionnaire. All of our patients including the pediatric subgroup, performed CIC by themselves without help of their caregivers. We found that 59% of the patients had no pain (the CIC wasn’t painful), 41% of the patients had conduit bleeding with catheterization. Eighty two percent hadn’t had residual pain after catheterization, 45.4% hadn’t had a blocking sensation with catheterization, although 41% despite it was infrequent (1 point) complained about it and 13.6% said they frequently (2 points) had a transitory blocking sensation. Eighty two percent hadn’t had a blocking sensation during catheter withdrawal (Table-2).

**Table 2:** Mitrofanoff Catheterization Difficulty Questionnaire (HUSI) - Adapted from the ICDQ and Global Satisfaction and Cosmetic Outcomes Questionnaire (HUSI).

Mitrofanoff Catheterization Difficulty Questionnaire (HUSI) - Adapted from the ICDQ					
	0 (Never) N (%)	1 (Infrequent) N (%)	2 (Frequent) N (%)	3 (Always) N (%)	Total (n)
1 - Do I have pain, or the CIC is painful?	13 (59)	7 (32)	1 (4.5)	1 (4.5)	22
2 - Does your stoma bleed with CIC?	13 (59)	9 (41)	0 (0)	0 (0)	22
3 - Do I have residual pain after the catheterization?	18 (82)	4 (18)	0 (0)	0 (0)	22
4 - Do I experience a blocking sensation and some force is required to insert the catheter?	10 (45)	9 (41)	3 (13.6)	0 (0)	22
5 - Do I have a blocking sensation during catheter withdrawal?	18 (82)	3 (13.6)	1 (4.5)	0 (0)	22
Global Satisfaction and Cosmetic Outcomes Questionnaire (HUSI)					
	Yes N (%)		No N (%)		Total N (%)
1 - Knowing what you already know, would you undergo a continent urinary diversion again?	16 (73)		6 (27)		22 (100)
2 - Are you satisfied with your continent urinary diversion?	20 (91)		2 (9)		22 (100)
3 - Would you recommend this type of reconstruction to a friend with your same problem?	21 (95)		1 (5)		22 (100)
4 - Are you satisfied with your body image when getting dressed?	17 (77)		5 (23)		22 (100)
5 - Are you satisfied with your stomal appearance?	14 (64)		8 (36)		22 (100)

Forty one percent of patient’s complaint of conduit bleeding infrequently and reported a blocking sensation while introducing the catheter, none of them had complaint about it in the follow-up visits despite having been asked. The blocking sensation while introducing the catheter could be explained as the sensation of over-coming the antireflux mechanism, because 82% did not have a blocking sensation during catheter withdrawal. Only 3 of 25 patients have had stomal stenosis and all cases resolved with conservative management.

Cosmetic and global satisfaction were assessed using a qualitative questionnaire designed by our group at the Reconstructive Urology Division of the HUSI. When asked, 73% of the patients would undergo CUD surgery with the Mitrofanoff principle again. A 91% satisfaction rate with their CUD was found. Ninety five percent of patients would not hesitate to recommend this kind of surgery to a friend or relative. Seventy seven percent of patients were satisfied with their body image when they dressed themselves and 64% were satisfied with their stomal appearance while 36% were unsatisfied (Table-2).

To evaluate differences in HRQoL, ease of catheterization, cosmetic and functional outcomes we performed a subgroup analysis comparing the eight patients with umbilical stoma against the seventeen patients with the VQZ-stoma. We found that patients in the umbilical stoma subgroup reported a mean EQ-VAS-score of 94+6.48 compared to a mean EQ-VAS-score of 83.6+14 in the VQZ-stoma. Difficulty with catheterization was similar in both groups. Satisfaction rates with the appearance of the stoma was lower in the umbilical stoma group without a clear explanation. Patients were more likely to have had an umbilical stoma when they were younger, no patient over 30 years-old at the time of surgery was chosen to have an umbilical stoma. Stomal stenosis or incontinence were complications not associated with the umbilical stoma group in our series.

## DISCUSSION

Few studies have evaluated HRQoL, ease of catheterization, cosmetic and functional outcomes of CUD with the Mitrofanoff principle. Despite its qualitative nature, our study is one of the most complete studies addressing this subject, with a long term follow-up, a wide variety of diagnosis and different stoma tailoring techniques.

CUD with the Mitrofanoff principle aims to create a continent catheterizable conduit that is easily accessible to the patient’s dominant hand (1, 4, 7, 13). Despite all the modifications of the technique, CUD with the Mitrofanoff principle remains a complex procedure with a significant non-negligible complication rate (14). This surgical reconstruction technique has been described for pediatric and adult patients (15).

Our study includes a wide range of age groups, as well as, diverse pathologies which demonstrates that this surgery is an important resource in any age group to resolve obstructive, functional and anatomic problems of the urinary tract. In the pediatric subgroup the mean age was 8.5 years, all patients were able to self-perform CIC and none of them have had stomal stenosis or another major complication.

There is scarce information about quality of life in this population. Smith, et al. evaluated 19 patients older than 16-years-old with the SF-36 health survey Version 2®. The score for Physical Functioning (PF=50.4), Role Physical (RP=53.8), Bodily Pain (BP=55.6), Vitality (VT=56.9), Social Functioning (SF=51.5), Role Emotional (RE=52.2), and Mental Health (MH=54.6) were all higher than those reported within the normal population (normal=50.0) and found the same results when compared against age-matched controls (9). This results are comparable with ours. We used a validated Questionnaire to assessed HRQoL, the EQ-5D-3L and the mean EQ VAS score was 86.5% which is comparable with the general population (8, 11). Lima, et al. evaluated HRQoL in patients with neurogenic bladder submitted to urological reconstructive surgeries: seven patients with cutaneous appendicovesicostomy, 1 continent cutaneous ileo-vesicostomy (Yang-Monti technique) and 1 patient with Malone antegrade continence enema (MACE) procedure. This study used the SF-36 Health Survey® and the Qualiveen® to measure patient reported outcomes measure (PROMs) and found improvement in all domains with statistical significance (8).

Few studies have evaluated ease or difficulty of catheterization in patients with CUD with the Mitrofanoff principle. Because of the lack of a validated questionnaire to assess difficult catheterization we decided to use the ICDQ questionnaire. It was developed by Guinet-Lacoste et al. in France, and validated in 70 patients with neurogenic bladder reliant on retrograde CIC (12).

They found a good internal correlation and a good test-retest correlation with an ICC of 0.81 and a cronbach alpha of 0.94 (12).

Self-perception and functional impact of the stoma is an important factor for children’s and adults. It is traditionally located at the right flank or the umbilicus and the stomal site and technique of construction has received as much attention and variation as the Mitrofanoff principle itself. Some authors suggest the best stoma tailoring technique is the VQ instead of the VQZ (16, 17). The latter results in a more prominent and irregular scar with the same complication rate or stoma continence (12, 14, 18, 19). In our study 17 stomas were tailored with the VQZ technique and 8 were umbilical stomas. Decisive factors for localization and type of stoma were length and mobility of the appendix mesentery. We found that 36% are unsatisfied with their stomal appearance, although we are under the impression that most of these patients were unhappy due to having to live with a stoma rather than with the cosmetic appearance of it.

Gowda, et al. evaluated the outcome of the Mitrofanoff stoma in 65 patients with a mean follow up of 75.2 months, 30 patients underwent a bladder-neck procedure at the same time of CUD. Difficulty catheterizing occurred in 46%, whilst 8% suffered stomal incontinence (18). Eleven stomal dilations and 38 skin level stomal corrections were performed. Overall, 97% of patients still had a catheterizable channel, which was continent in 95% (18). Sahadevan, et al. audited the long-term outcome of 29 adult patients with a Mitrofanoff CUD, with a mean follow-up 126 months. Of those 71% had an appendicovesicostomy and the remaining an ileal conduit (Yang-Monti). An 89% continence rate was reported. Stomal stenosis occurred in 54% of the stomas. Conversion to an ileal conduit was required in 18% of patients, two for persistent incontinence and three for recurrent stomal complications (19). In our study we found a 12% stomal stenosis rate and 100% were continent after bulking agent injection in one patient, and bladder neck closure in another one. One of the longest follow-up series in CUD with the Mitrofanoff principle was presented by Liard, et al., with a 20-year follow-up of their patients. This study showed that 16 of 23 patients still had a catheterizable and continent stoma and 9 patients had stomal stenosis despite all efforts to prevent it (20).

Despite all efforts in modifying the technique, stomal stenosis remains the most common complication reported in the literature, with a rate of stenosis of 6 to 39%. We found a 12% rate of stomal stenosis, all successfully managed with conservative measures and all of the stomal stenosis complications in the subgroup of the flank VQZ stoma. Other more serious complications where partial and total necrosis of the appendiceal conduit, retrospectively we think the length of the appendix mesentery wasn’t enough for building the catheterizable conduit. These complications have already been reported in other series but every effort should be made to prevent them (4, 14).

The limitations of our study are the cross-sectional design and retrospective nature of it, as well as reporting outcomes of pediatric and adult patients. Some of the questionnaires were not designed or validated in pediatric population but given the heterogeneity of our population we decided to use one questionnaire for evaluating HRQoL. A prospective design measuring PROMs before and after surgery would help to resolve many questions remaining in the CUD surgery.

## CONCLUSION

Continent urinary diversion with the Mitrofanoff principle is associated with a good HRQoL, ease of catheterization in most patients, remarkable global satisfaction with the procedure and adequate self-perception of the cosmetic results. It remains a safe and viable option for children and adults who have the indication, with a low complication rate and need for re-intervention. The umbilical stoma patients had better quality of life scores compared with V-Quadrilateral-Z (VQZ) plasty, despite having lower satisfaction rates with the appearance of the stoma. Stomal stenosis or incontinence were complications not associated with the umbilical stoma group in our series, despite being reported on other articles.
